# Lessons in longevity from blood stem cells under protein stress

**DOI:** 10.1016/j.tcb.2025.06.006

**Published:** 2025-07-29

**Authors:** André Catic

**Affiliations:** 1 Department of Molecular and Cellular Biology, Huffington Center on Aging, Stem Cells and Regenerative Medicine Center, Dan L. Duncan Comprehensive Cancer Center, Baylor College of Medicine, Houston, TX, USA; 2 www.caticlab.org

## Abstract

Blood stem cells are among the body’s longest-living cells despite being highly vulnerable to proteotoxic damage, which accelerates their aging. To maintain protein homeostasis (proteostasis), hematopoietic stem cells (HSCs) employ mechanisms such as reduced translation rates, high chaperone activity, autophagy, and selective protein degradation. These strategies mitigate protein misfolding, maintain quiescence, and preserve regenerative potential. Disruptions in proteostasis can lead to the elimination of impaired HSCs through differentiation or apoptosis, ensuring the integrity of the stem cell pool. Due to the systemic impact of the blood on aging and its experimental and clinical accessibility, investigating HSC proteostasis provides insights into longevity and potential therapeutic strategies. This review examines emerging mechanistic links between proteostasis and HSC fate, concluding with unresolved questions and challenges of the current research.

## Quiescence enables maximum lifespan through minimal activity

Protein stress is intricately linked to aging through the accumulation of damaged and misfolded proteins, which gradually compromise cellular function and viability. Reducing protein translation is a central theme across diverse longevity interventions. Approaches such as caloric restriction or rapamycin extend lifespan by alleviating proteotoxic load. As described in this review, blood stem cells have uniquely tuned proteostatic mechanisms to avoid the accumulation of misfolded proteins with age. Given the growing aging population and the increasing prevalence of age-associated diseases, understanding these mechanisms has become a priority.

Adult stem cells in blood, muscle, brain, mesenchyme, and hair follicles maintain a reversible quiescent state marked by low metabolism and reduced protein synthesis, preserving long-term regenerative potential [1–3]. During quiescence, proteostasis networks clear damaged proteins to maintain a high-fidelity proteome for rapid activation upon injury [1,4]. By contrast, embryonic and induced pluripotent stem cells exhibit continuous proliferation and high translation rates, and rely on robust chaperones and proteasomes to sustain pluripotency, highlighting a fundamental difference from the restrained and damage-avoidant nature of adult stem cell quiescence [3,4].

Misfolded protein accumulation—a by-product of translation—drives morbidity and mortality and is a hallmark of aging [5–7]. The central nervous system is especially vulnerable, while the blood remains relatively resilient despite containing long-lived HSCs that can outlive their hosts in experimental and clinical transplantation settings. This resilience is notable given the constant regeneration that hematopoiesis demands. HSCs withstand persistent stress from DNA damage, protein misfolding, metabolic shifts, and microenvironmental changes [8–16]. While progenitors proliferate, HSCs stay largely quiescent, activating only under stress. This reversible dormancy preserves function but requires tight control. The tension between quiescence, regeneration, and protein stress management is a core challenge in HSC biology.

Recent evidence highlights that aged HSCs can suffer from protein aggregation similar to that in post-mitotic neurons. The prion-like protein FUS, known for forming insoluble aggregates in neurodegenerative diseases such as frontotemporal dementia and amyotrophic lateral sclerosis (ALS) [17], also forms viscous condensates in aged HSCs under physiological conditions. These aggregates are primarily nuclear in HSCs and compromise chromatin organization, contributing to HSC dysfunction [18]. It is likely that additional forms of proteinopathy exist within the aged hematopoietic system; this awaits future discovery.

**Figure 1.**
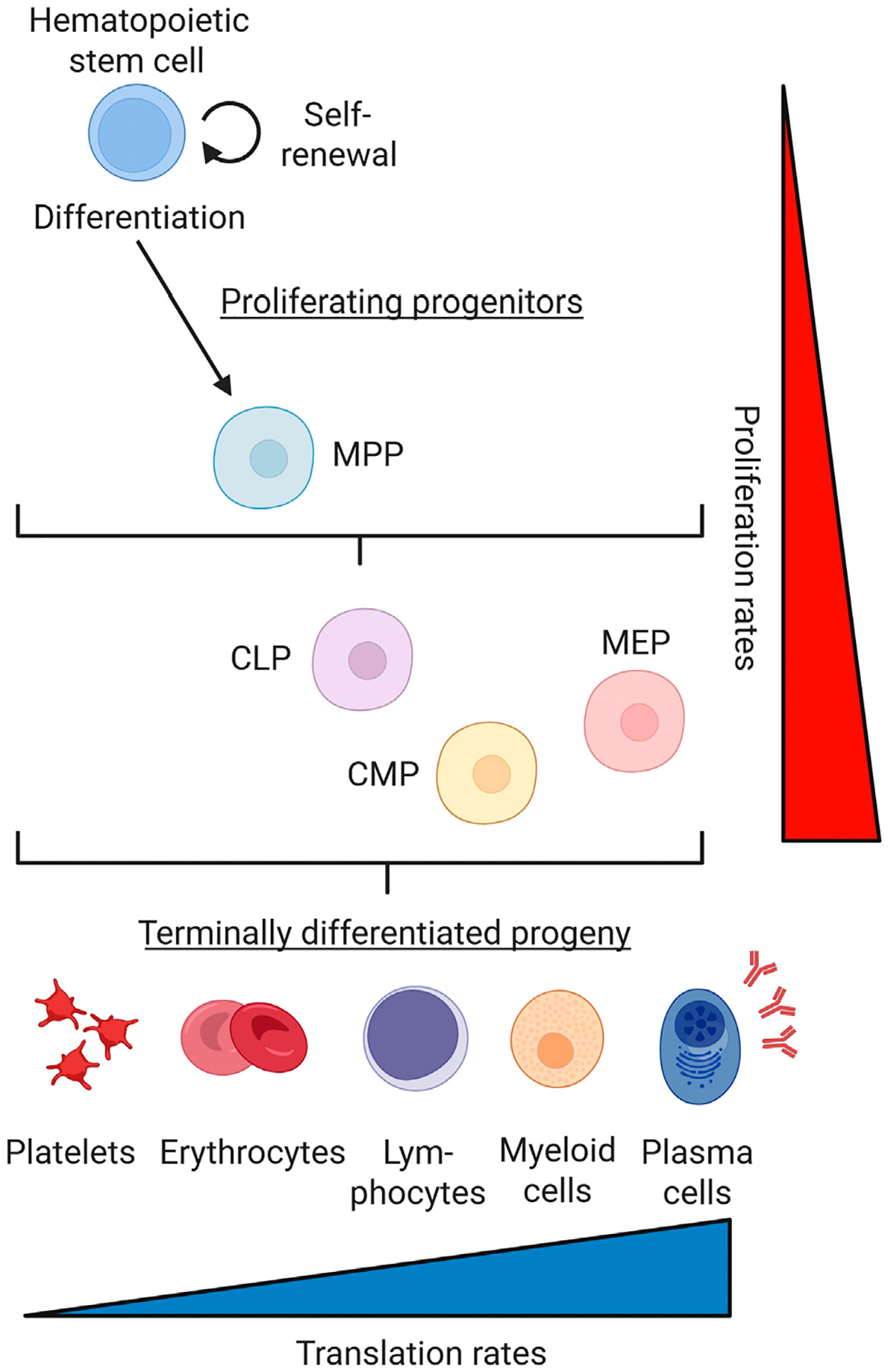
Schematic overview of hematopoiesis. Illustrating the differentiation hierarchy from quiescent hematopoietic stem cells (HSCs) to proliferating progenitors—multipotent progenitors (MPPs), common lymphoid progenitors (CLPs), common myeloid progenitors (CMP), and megakaryocyte-erythroid progenitors (MEP)—and ultimately to terminally differentiated blood cells. HSCs maintain the blood system by balancing self-renewal and differentiation. HSC divisions can result in the retention of stem cell properties or generation of progenitor cells with lineage bias. New studies indicate that rather than strict asymmetric outcomes, cellular fate potential exists in a continuum [31,32]. Under steady-state conditions in mice, most blood cells are produced by actively cycling progenitor cells, while HSCs contribute minimally to mature blood cell production. In response to stress, HSCs become more active, which may accelerate differentiation and increase the risk of stem cell exhaustion. Protein homeostasis demands vary widely across blood lineages: red blood cells eliminate their ribosomes and largely cease protein synthesis, while plasma cells are among the most prolific protein-producing cells in the body [39,137,138]. Translation and proliferation rates are not drawn to scale. Translation rates in stem and progenitor cells are depicted independently from those in terminally differentiated progeny, which reflect non-dividing cells. Figure created with BioRender.

Adding complexity, the hematopoietic system spans a wide spectrum of protein synthesis demands: from anucleate red blood cells with little to no translational activity to plasma cells that rank among the highest protein-producing cells in the body (Figure 1). This range makes hematopoiesis an ideal model to study context-specific proteostasis. The clinical relevance of this axis is highlighted by ribosomopathies: rare congenital disorders of ribosome biogenesis that, despite affecting a ubiquitous cellular process, manifest with striking blood-specific phenotypes [19,20]. Hematopoiesis relies heavily on functional ribosome assembly due to its high regenerative demand and structural challenges in translating key hematopoietic factors [21]. Evolutionary pressures to bypass ribosomal quality control predispose patients with ribosomopathies to leukemias, underscoring the critical role of translational regulation in both normal and malignant hematopoiesis.

Age-related immune decline accelerates organismal aging, and HSCs may contribute to this process. While blood appears resilient under steady-state conditions, HSCs are paradoxically vulnerable to proteotoxic stress [22]. This vulnerability is functionally masked by rapidly dividing progenitor cells that are responsible for homeostatic hematopoiesis, but emerges during stress, such as inflammation or post-transplant repopulation, when HSCs must proliferate [23]. To preserve function, HSCs rely on tightly regulated proteostasis pathways, including autophagy, mitophagy, and the ubiquitin–proteasome system. These decline with age, reducing lymphoid output and immune resilience. Given the immune system’s body-wide surveillance role, its dysfunction has broad consequences. T cell defects accelerate aging in multiple tissues [24,25], and clinical studies suggest that impaired lymphopoiesis late in life increases susceptibility to age-associated diseases [26].

Proteostasis failure in HSCs has been linked to downstream immune dysfunction. Deficits in mitophagy and mitochondrial quality that originate in HSCs can be inherited by lymphoid cells, suggesting that early HSC defects may propagate through the hematopoietic hierarchy [27,28]. Another example is VEXAS syndrome—a non-canonical form of clonal hematopoiesis in humans—where somatic mutations in the ubiquitin-activating enzyme UBA1 lead to proteostatic collapse in HSCs, skewed hematopoiesis, and widespread immune dysfunction [29,30]. Although classical clonal hematopoiesis typically does not involve proteostasis genes, additional non-canonical forms may exist in which disrupted protein homeostasis gives rise to maladaptive clones that expand in an inflammatory environment, outcompeting the healthy HSC pool.

**Figure 2.**
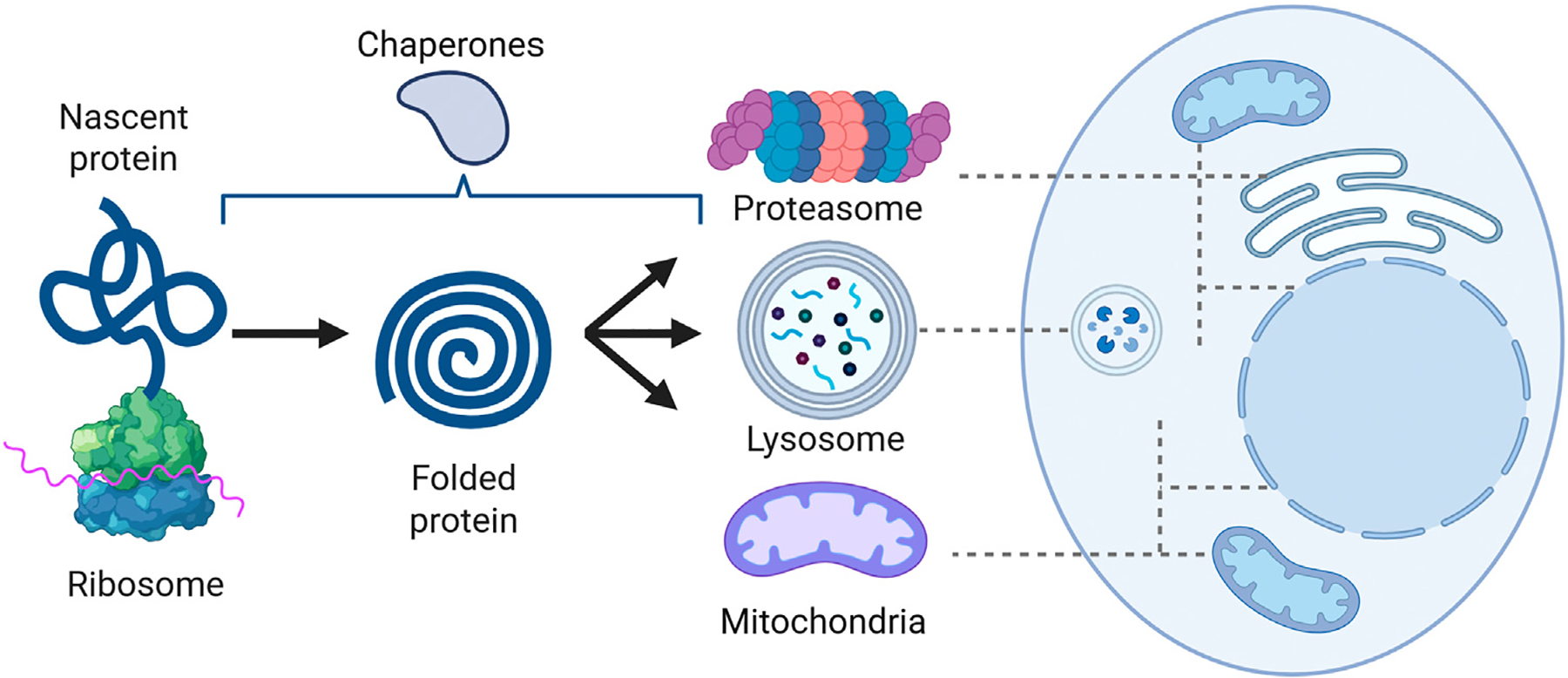
Life cycle of proteins. Proteins encounter several key decision points during their lifespan. Blood stem cells tune protein abundance not only by regulating gene expression but also by modulating translation rates. Molecular chaperones assist in the proper folding of nascent peptide chains and support protein stability and function throughout their lifetime. Once a protein has completed its functional life cycle or becomes damaged, it is targeted for removal. The two primary pathways for complete protein degradation are the lysosomal pathway—operating in the cytoplasm—and the proteasomal pathway, which functions in both the cytosol and nucleus, but which can also selectively extract damaged proteins from mitochondria and the endoplasmic reticulum. Although many other proteases exist within cells, most are involved in processing or trimming proteins rather than fully degrading them. Exceptions are mitochondrial proteases, which can fully degrade proteins within mitochondria, and which have also been implicated in the degradation of extramitochondrial proteins [59–61]. Figure created with BioRender.

Powerful experimental techniques such as transplantation and parabiosis have been employed to study protein stress in hematopoiesis. At the same time, these approaches need to be carefully evaluated, given that *in vitro* cultures and *in vivo* manipulations often result in accelerated differentiation, impairing our ability to critically study the role of proteostasis within the quiescent HSC subpopulation [31,32]. Given the conserved nature of proteostasis mechanisms across tissues, insights gained from blood stem cell proteostasis are likely broadly applicable [33,34]. This review focuses on how various protein quality control mechanisms influence HSC aging, ultimately ensuring their long-term functionality (Figure 2).

## Controlling translation

Low translation rates are essential for stem cell longevity [22,35–38]. Increasing ribosome activity shortens lifespan, suggesting that dormant stem cells poorly tolerate proteotoxic stress. HSCs limit translation through reduced transcript levels, including ribosomal subunits [39], and strict regulation of eIF4E, the cap-binding factor that initiates the bulk of translation [40]. Also, mRNA abundance and translation are highly uncoupled in HSCs [39,41], implying that mRNA presence does not guarantee protein production. Emerging research in epitranscriptomics is exploring how modifications such as m6A affect mRNA stability and translation, supporting stem cell quiescence [42].

A pivotal regulator of stem cell fate is c-MYC, a master transcription factor that drives the expression of genes involved in protein translation. Elevated c-MYC promotes proliferation and can trigger differentiation [43–45]. While c-MYC is essential for development and repair, reduced levels of this transcription factor can extend the lifespan in mice and improve the function of several organs under controlled laboratory conditions [46]. Together, these findings illustrate that c-MYC–driven control of translation is not merely a downstream consequence of developmental cues, but may actively control the balance between quiescence and differentiation. The role of c-MYC inheritance during (a)symmetric cell division is an important area of ongoing investigation [47].

Translational efficiency in HSCs is also limited by the initiation factor eIF2. When the α subunit is phosphorylated, eIF2 reduces global translation while favoring synthesis of stress-response proteins such as ATF4. This phosphorylation is regulated by the integrated stress response (ISR), which remains tonically active in HSCs even under basal conditions [48,49]. Under severe proteotoxic stress, HSCs undergo rapid apoptosis, a protective mechanism that prioritizes stem cell quality over quantity [50]. Indeed, selectively removing functionally old HSCs is now being explored to rejuvenate the stem cell population, even though it reduces the overall HSC number [51]. The ISR also includes two organelle-specific arms: the unfolded protein response (UPR) in the endoplasmic reticulum (ER) and the mitochondrial unfolded protein response (UPRmt). Both converge on eIF2 phosphorylation but also have organelle-specific effects, such as inducing chaperones tailored to the stressed compartment. Mitochondria, in particular, represent a harsh biochemical environment that can promote protein damage and misfolding. Besides generating reactive oxygen species and operating at temperatures up to 50°C, their high pH can deprotonate lysine side chains, promoting spontaneous acetyla tion [52,53]. Because mitochondria have a high concentration of acetyl groups, these modifications occur frequently, requiring deacetylases such as sirtuins to remove them. Studies show that the mitochondrial sirtuin Sirt3 regulates HSC fate [9,54], whereas the nuclear enzyme Sirt7 promotes HSC quiescence by repressing mitochondrial genes [55]. Sirtuins require NAD^+^ as a cofactor, which may explain the compound’s beneficial effects in several longevity studies [13,56,57]. Proteases are also critical to maintain mitochondrial activity under these harsh conditions [58]. In addition, emerging data suggest that mitochondrial proteases, including those governing the UPRmt, may act on substrates beyond their immediate mitochondrial environment, potentially influencing cytosolic and nuclear proteins. Although the mechanisms remain unclear, such extramitochondrial effects may help coordinate global proteostasis in response to mitochondrial stress [59–61].

Beyond controlling the transcription and modification of RNA, HSCs can limit protein synthesis by restricting mRNA access to ribosomes. They do so by sequestering transcripts in processing bodies (P-bodies), cytosolic granules that withhold mRNAs from translation. Under homeostatic conditions, P-bodies are dispensable [62], but during regenerative stress—such as post-transplantation—HSCs rely on these stores, underscoring the role of mRNA sequestration in proteostatic regulation.

Emerging research highlights that ribosomes function not only as translational machines but also as key sensors and modulators of cellular stress, viscosity, and molecular crowding [63,64]. Through this capacity, ribosomes help integrate environmental and metabolic cues with translational control, thereby influencing stem cell fate decisions. In this context, ribosomal stress is increasingly recognized as a potential contributor to HSC aging. Osmotic stress or ribosomal collisions on mRNAs can trigger stress responses that slow proliferation or induce apoptosis. Because these stresses become increasingly relevant with age, they may be critical in stem cells that must remain functional throughout the organism’s lifespan. HSCs, with their small cytoplasmic volume, may be particularly susceptible to ribosomal crowding. This heightened sensitivity could be a contributing factor to why ribosomopathies—primarily germline mutations in ribosomal components that affect all tissues—often manifest with pronounced hematopoietic defects [65]. In addition, new studies have discovered that genotoxic insults not only cause DNA damage, but also result in RNA damage that causes ribosomal collisions, triggering a ribotoxic response [66]. Understanding how ribosomes and ribosomal stress serve as sensors to specifically influence blood stem cell fate and aging is an exciting topic for future research.

Ribosome assembly begins in the nucleolus, a large phase-separated nuclear body. While primarily known for rRNA transcription, the nucleolus also functions as a stress sensor and protein folding site [67,68]. Its activity adapts to genotoxic stress via p53 and responds to nutrient levels. Further, smaller nucleoli correlate with longer lifespan, linking nucleolar function to metabolic and proteostatic control [69,70]. In aged HSCs, nucleolar stress reduces translation, potentially preserving longevity at the cost of diminished differentiation capacity [71,72].

In summary, protein synthesis in HSCs is regulated at multiple levels: from ribosome biogenesis, the initiation steps of cap-dependent translation, and the accessibility of readable mRNA, to the sensitivity of both global and organelle-specific stress–response pathways. Moreover, recent discoveries indicate that ribosomes play roles beyond protein production, potentially guiding HSC fate decisions.

## Supporting protein folding

As soon as a protein exits the ribosome, it is immediately guided by various chaperones. Humans express around 330 chaperones nearly ubiquitously across tissues [73]. Although the overall proteome can differ by up to 50% between cell types, chaperones typically show only about 10% variation in expression, reflecting their fundamental role in protein homeostasis. Nevertheless, the mix of chaperones at work and the conditions under which they function can be cell-type-specific and emphasize, for instance, support of secretory pathways in antibody-producing plasma cells [74,75].

Ribosome-associated chaperones help ensure that the substantial ATP and biomolecule investment in protein production is not wasted [76]. Much of our understanding of early protein folding stems from bacterial studies of trigger factor, a dedicated ribosome-associated chaperone. Trigger factor binds nascent peptide chains via a cradle-like ‘holdase’ domain and includes a ‘foldase’ domain with prolyl isomerase activity [77]. Proline is unique among amino acids in adopting either a *trans* (more common) or *cis* configuration, a transition catalyzed by prolyl isomerases. In mammals, the prolyl isomerase Cyclophilin A appears to mirror trigger factor, exhibiting dual holdase and foldase activity [78] and associating with ribosomes [79]. Prolyl isomerization can be rate-limiting for protein synthesis [80,81]. In hematopoietic stem and progenitor cells, Cyclophilin A is highly abundant, comprising over 10% of the cytosolic proteome. Its absence accelerates stem cell aging, while overexpression improves the function of aged HSCs [82]. A proteomic analysis of Cyclophilin A’s client proteins revealed enrichment for DNA/RNA-binding proteins with intrinsically disordered regions. Several of these undergo liquid–liquid phase separation, forming membrane-less bodies via demixing from the surrounding environment. Prolyl isomerization has been proposed to regulate the formation and dissolution of such condensates. By acting early during translation, Cyclophilin A may shape the physical properties of nascent proteins and influence their ability to phase-separate. Intrinsically disordered proteins translate more slowly than structured ones [76,82], suggesting that their synthesis may depend on slow-acting chaperones such as prolyl isomerases. In this context, it is remarkable that HSCs show a profound loss of soluble disordered proteins alongside an age-related decline in Cyclophilin A levels—an effect similarly observed in Cyclophilin A knockout models [82]. In contrast to the age-related decline in Cyclophilin A, heterochronic parabiosis—exposing young mice to old blood—leads to a strong upregulation of this chaperone, potentially reflecting a response to aging-associated stress, though its impact on regenerative capacity is not yet fully understood [83–85]. Determining whether proteome-wide changes in structural properties affect the cellular capacity to form membrane-less organelles with age is an important focus for future research.

The role of biomolecular condensation is increasingly recognized in hematopoiesis, linking phase-separated compartments to stem cell fate and immune function [62,71]. Biomolecular condensates such as nucleoli, stress granules, and P-bodies compartmentalize cellular processes by selectively enriching or excluding specific macromolecules [71,86]. However, several caveats must be considered when studying liquid–liquid phase separation. Overexpression systems often produce artificial condensates due to non-physiological protein levels, leading to uncontrolled aggregation. Pharmacological tools such as 1,6-hexanediol, commonly used to disrupt condensates, have broad, off-target effects that complicate interpretation. Visualizing cytoplasmic phase separation in primary HSCs is technically challenging due to their minimal cytoplasmic volume, making it difficult to detect structures such as stress granules. By contrast, nuclear condensates such as nucleoli remain observable [82]. Overcoming these limitations through improved physiological models, endogenous labeling, and advanced microscopy will be critical for defining the functions and regulation of phase separation in HSC biology.

Another key chaperone-mediated mechanism is the heat shock response (HSR), a conserved program that promotes protein quality control by inducing chaperones, particularly important in aging HSCs. HSF1, the master regulator of the HSR, activates heat shock protein (HSP) expression to support protein folding, prevent aggregation, and maintain cellular integrity, thereby enhancing HSC resilience and longevity. Protein synthesis rates change with age [87,88], compounding proteotoxic stress. HSR activation helps counteract this by coordinating chaperone expression to mitigate misfolding and preserve HSC function [89]. Kruta *et al*. showed that HSF1 is dispensable in young adult HSCs *in vivo* but becomes upregulated in aged HSCs, where it suppresses excessive protein synthesis and prevents proteotoxicity [89]. This is surprising because HSF1 is typically associated with conditions of elevated translation, functioning in co-ordination with mammalian target of rapamycin (mTOR) signaling to support the folding of nascent proteins—rather than with the ISR, which reduces translation [90]. Thus, in hematopoiesis, the HSR may play an unconventional role akin to that of the ISR: supporting proteostasis not only by inducing chaperones but also by limiting bulk protein production. The study also found that *ex vivo* culture of HSCs induces a dramatic rise in protein synthesis, underscoring the disconnect between *in vitro* and *in vivo* conditions. This raises concerns about overinterpreting results from cultured HSCs, which differ markedly from their native, quiescent, and translationally repressed state in the bone-marrow niche.

## Enforcing protein removal

**Figure 3.**
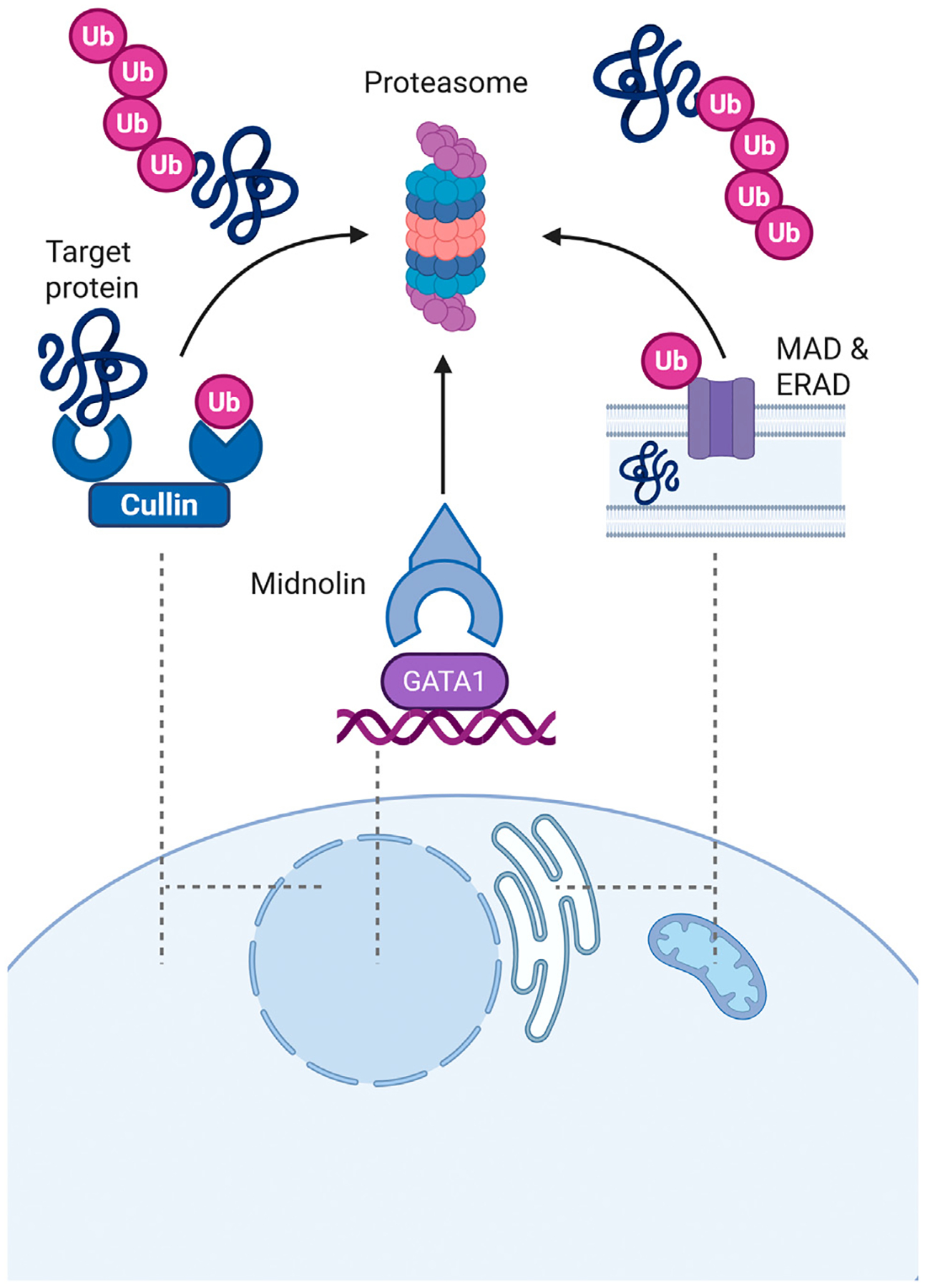
Overview of the ubiquitin–proteasome system (UPS). The proteasome, a multi-subunit protease, operates in both the cytosol and the nucleus, predominantly degrading proteins tagged with ubiquitin (Ub). Cullin-RING ubiquitin ligases, the largest family of E3 enzymes, selectively recognize and ubiquitinate a diverse array of substrates due to the modular structure of the cullin complexes. Misfolded proteins from the endoplasmic reticulum (ER) and mitochondria can be extracted via ER-associated degradation (ERAD) and mitochondria-associated degradation (MAD) pathways, respectively, for proteasomal degradation in the cytosol. Additionally, the proteasome is capable of degrading certain proteins in a ubiquitin-independent manner. For instance, the receptor protein midnolin can selectively recognize several transcription factors. Figure created with BioRender.

Proteins that misfold or reach the end of their lifespan must be degraded. The two primary pathways for protein disposal involve proteasomes and lysosomes. The proteasome mediates most selective, protein-specific turnover through the ubiquitin–proteasome system (UPS): a dedicated pathway that marks proteins for degradation. The UPS involves a cascade of enzymes that covalently attach ubiquitin to target proteins (Figure 3). This includes two activating enzymes, several dozen conjugating enzymes, and hundreds of ubiquitin ligases that confer substrate specificity [91,92]. This review highlights two major ways in which selective protein removal by the UPS regulates HSCs.

**Figure 4.**
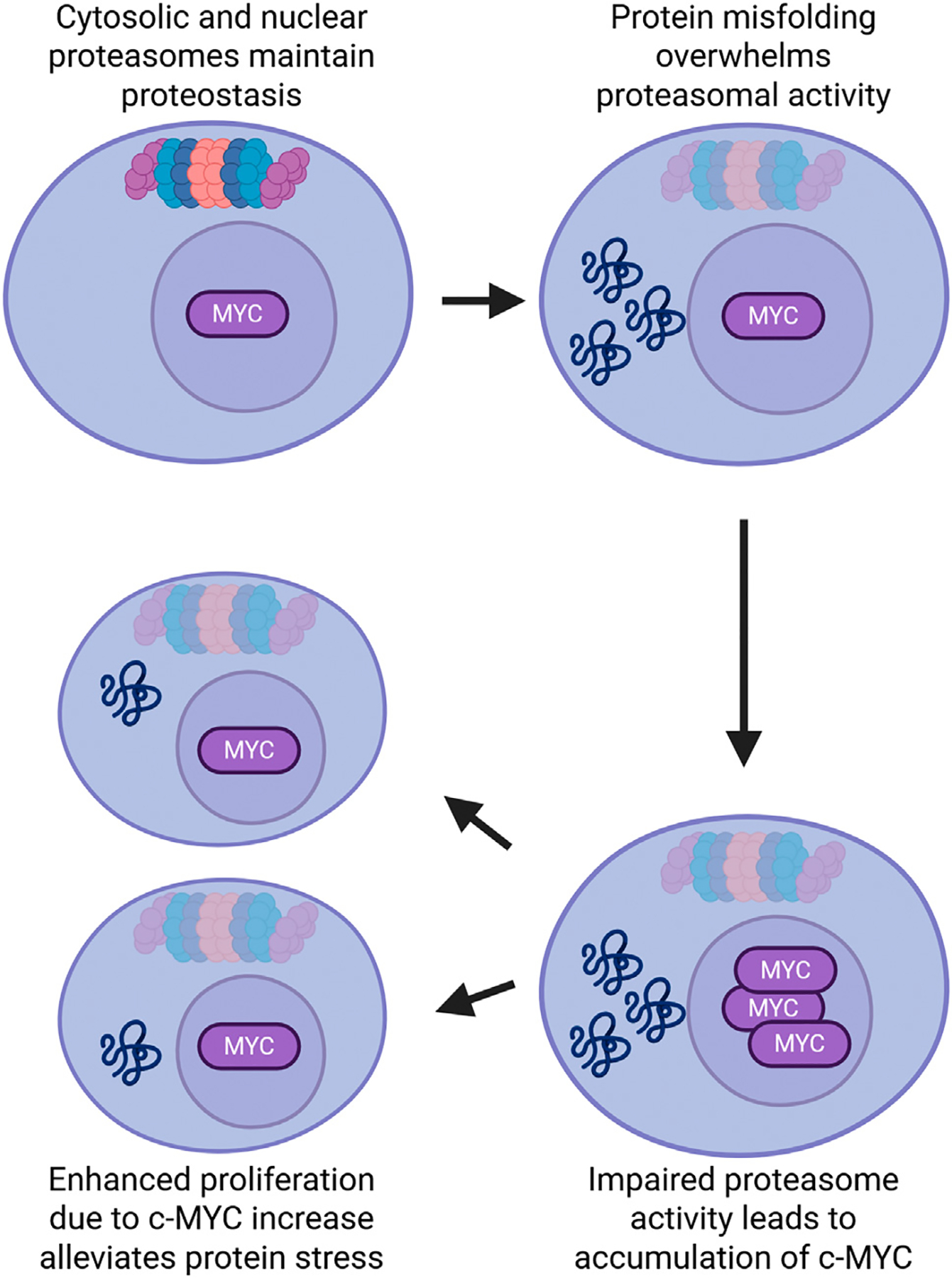
The c-MYC overflow model. Impaired protein degradation by the proteasome stabilizes the otherwise short-lived transcription factor c-MYC, driving cell division in the HSC compartment. While this mechanism may temporarily reduce the burden of protein aggregates through dilution, it ultimately depletes the pool of quiescent stem cells and can lead to HSC exhaustion [22]. Figure created with BioRender.

As noted, c-MYC is a master regulator that drives HSCs from quiescence into proliferation and, in some contexts, differentiation. As a transcription factor, c-MYC promotes genes involved in growth, energy production, and cell division. However, both c-MYC protein and mRNA are highly unstable, requiring constant synthesis. Multiple ubiquitin ligases target c-MYC, many of which are expressed in hematopoietic cells [93,94]. Low c-MYC levels are critical for maintaining HSC quiescence. A drawback of dormancy is that, like post-mitotic cells such as neurons, HSCs cannot dilute protein aggregates through division. When proteotoxic stress increases and the UPS fails to degrade c-MYC, it accumulates, triggering proliferation [22]. In this context, elevated c-MYC may act as a ‘release valve’, enabling cell division to dilute misfolded proteins at the cost of quiescence (the c-MYC overflow model) [22] (Figure 4). Moreover, forced proliferation can push proteostasis-impaired cells into irreversible differentiation, preserving the integrity of the quiescent stem cell pool. The UPS is less active in quiescent HSCs, which may explain why the HSC pool expands with age despite declining regenerative potential: impaired proteostasis stabilizes c-MYC, nudging HSCs toward proliferation and reducing their quiescence-dependent long-term function while increasing short-term cell numbers.

A second distinctive UPS function in HSCs involves the ER, the cell’s main lipid-organizing center and a site of protein production for membranes and organelles. The ER-associated degradation (ERAD) pathway clears misfolded proteins from the ER through extraction and proteasomal degradation, preventing defective proteins from reaching the plasma membrane or intracellular vesicles. Whether dormant or active, HSCs rely on surface receptors to communicate with the environment and respond to situations such as blood loss or inflammation [95,96]. A critical plasma membrane receptor is MPL (myeloproliferative leukemia protein), the thrombopoietin receptor that supports HSC quiescence. When ERAD is impaired, MPL misfolds and is retained in the ER instead of reaching the cell surface [97]. Loss of MPL leads to stem cell exhaustion and impaired regeneration. ERAD also modulates lysosomal function by regulating protein quality in the ER and its downstream vesicles. Lysosomes are not just degradation sites, they recycle biomolecules and act as nutrient-sensing platforms. The mTOR complex, a key metabolic regulator, localizes to the lysosomal membrane. ERAD influences this system by controlling levels of Rheb, a lysosome-associated GTPase that activates mTOR [98]. By fine-tuning Rheb abundance, ERAD links protein quality control to nutrient sensing and metabolic regulation essential for stem cell maintenance.

C-MYC degradation and ERAD typically require ubiquitination before proteasomal destruction, but the proteasome can also degrade proteins without prior ubiquitin tagging. This less understood mode of substrate recognition is emerging as a critical regulatory layer in cell biology, particularly in controlling transcription factor turnover. One key mediator of ubiquitin-independent degradation is Midnolin, a protein initially discovered in *Drosophila melanogaster* as Stuxnet. This factor acts as a target recognition motif that delivers substrates directly to the proteasome without previous ubiquitination [99,100]. Midnolin/Stuxnet is particularly intriguing because it explicitly targets transcription factors relevant to HSC quiescence and hematopoiesis, including EGR1 [101], IRF4 [102], and GATA1 [103]. Further, Midnolin has been identified as a genetic risk factor for neurodegeneration [104], potentially through EGR1 accumulation, and it is tempting to speculate that impaired ubiquitin-independent turnover of transcription factors could also contribute to age-related anemia or immune dysfunction.

**Figure 5.**
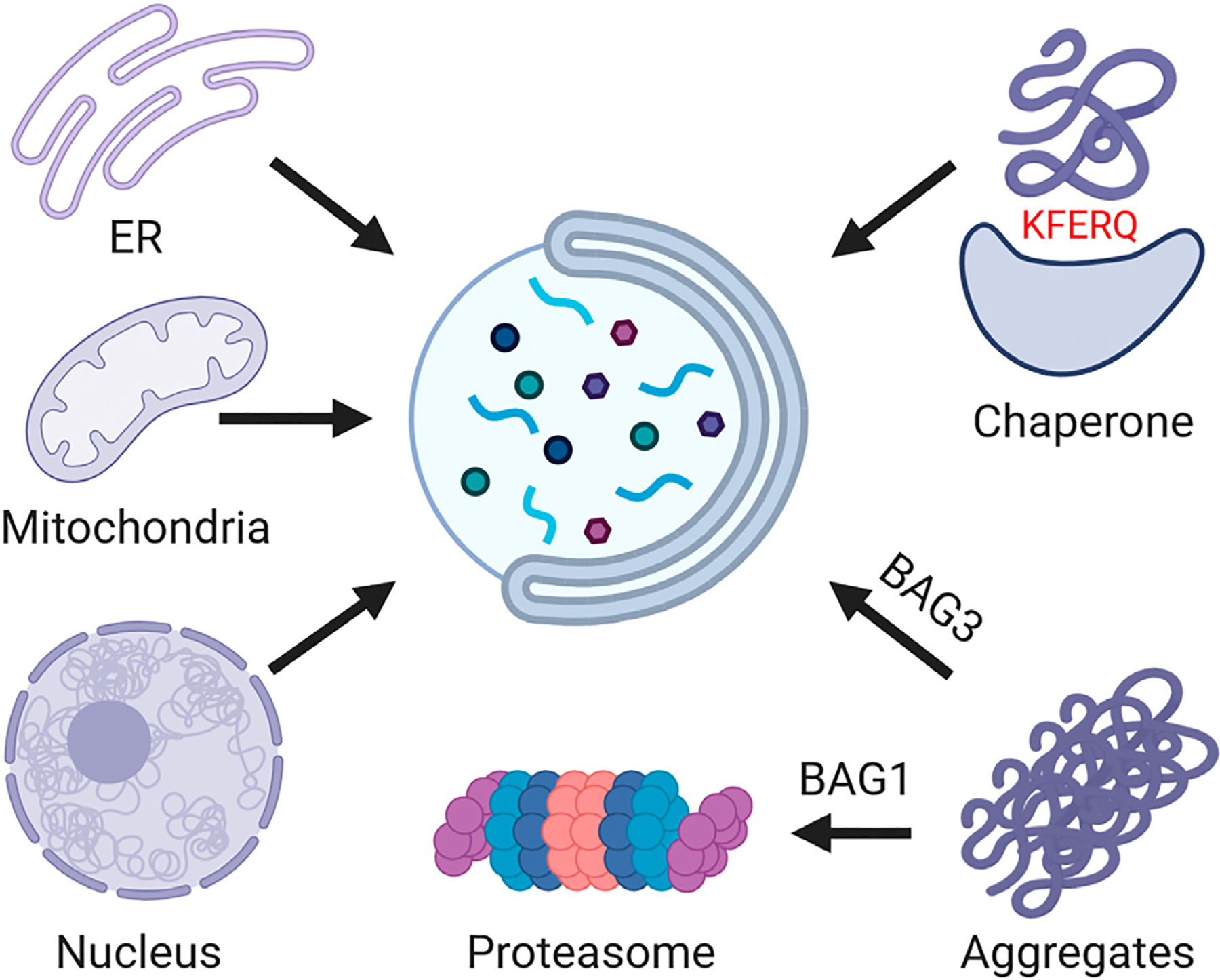
Overview of lysosome-dependent proteostasis. Macroautophagy is responsible for the bulk or partial degradation of organelles: including the endoplasmic reticulum (ER), nuclear components, and especially mitochondria. In doing so, this pathway modulates cellular metabolic states and removes damaged organelles. Aggregates of misfolded proteins can be cleared via two distinct routes: smaller aggregates or individual misfolded proteins may be degraded by the proteasome with the assistance of the BAG1 co-chaperone. Larger protein aggregates are directed toward autophagic degradation through the action of the BAG3 co-chaperone, a process known as chaperone-assisted selective autophagy (CASA). These aggregates are first organized into aggresomes—perinuclear structures encased in vimentin filaments—which are then engulfed by autophagosomes for degradation. Chaperone-mediated autophagy (CMA) selectively targets individual proteins bearing a KFERQ-like pentapeptide motif, facilitating their direct translocation into lysosomes. Figure created with BioRender.

While the proteasome degrades individual misfolded proteins, the autophagy pathway is mainly responsible for the bulk removal of protein aggregates and entire organelles (Figure 5). In HSCs, selective autophagy of mitochondria (mitophagy) is essential for maintaining quiescence [105]. Mitochondria provide the energy and biosynthetic precursors required for cell proliferation, yet quiescent HSCs maintain a metabolically dormant state characterized by low mitochondrial activity and reduced glycolysis [88,106–108]. Activation of mitochondrial function can drive HSCs into the cell cycle—likely via mTOR signaling—underscoring the importance of suppressing mitochondrial activity to preserve long-term quiescence [109]. Indeed, a subset of quiescent HSCs with high mitophagy exhibits the greatest long-term repopulation potential, even as they age [88]. Moreover, another study revealed that in quiescent HSCs, mitochondria can be sequestered in unacidified lysosomal vacuoles, where they are not immediately degraded. Upon activation, these vesicles acidify to digest and recycle mitochondrial components, generating substrates and energy for proliferation and differentiation [106]. Adding to this complexity, a third study found that aged HSCs can harbor large numbers of mitochondria that may be functionally repressed, suggesting an additional, though less well-defined, mechanism for restraining mitochondrial activity [110].

In addition to clearing damaged organelles, autophagy also eliminates misfolded proteins, especially when these proteins are sequestered into organized structures called aggresomes. In young HSCs, aggrephagy (the selective autophagic removal of aggresomes) serves as the primary pathway for disposing of misfolded proteins, which is crucial given that these cells exhibit relatively low proteasome activity [111]. However, as HSCs age, autophagic flux declines while UPS activity increases. Aged HSCs display a profound loss of aggresomes. Whether this decline in aggresome formation is due to enhanced compensation by the UPS, an intrinsic inability to form aggresomes, or reduced synthesis of aggregation-prone intrinsically disordered proteins with age [82], remains to be determined.

By contrast with bulk autophagy or aggrephagy, chaperone-mediated autophagy (CMA) selectively degrades individual soluble proteins that contain a pentapeptide motif. In this pathway, the chaperone HSC70 recognizes the motif and delivers the substrate to lysosomes via the receptor LAMP-2A [112]. In HSCs, CMA is upregulated upon activation and plays a key role in metabolic reprogramming by clearing damaged or inactive enzymes to enhance fatty acid oxidation. CMA activity declines with age, contributing to impaired proteostasis and metabolic dysfunction in aged HSCs. Boosting CMA by stabilizing components such as HSC70 and LAMP-2A has been shown to restore metabolic homeostasis and improve the self-renewal capacity and function of older HSCs.

Overall, autophagy declines in aging HSCs [112,113], although a subset of HSCs retains a ‘fit’ or functionally robust autophagic state [88]. UPS activity and chaperone expression tend to increase with age [38], possibly as a compensatory mechanism for the reduced autophagic flux. Quiescent HSCs rely more heavily on autophagy for protein quality control, and new evidence indicates that the asymmetric inheritance of lysosomes during HSC division can bias one daughter cell toward activation and differentiation, while preserving a pool of quiescent stem cells [114].

## Enhancing protein homeostasis

Among interventions aimed at preserving proteome integrity in aging cells, autophagy activators have shown considerable promise. Rapamycin, an mTOR inhibitor that enhances autophagocytosis, can partially rejuvenate HSC function [109,115]. However, its inherent immunosuppressive effects are particularly concerning in older individuals. A more physiological alternative is caloric restriction, which also downregulates mTOR signaling, thereby reducing translation rates and promoting autophagy. Even short-term dietary interventions can boost HSC repopulation capacity, though these benefits involve multiple factors—including metabolic reprogramming—and cannot be attributed solely to changes in proteostasis [113].

An evolutionarily conserved strategy to manage protein stress—also observed in bacteria—involves asymmetric cell division, in which damaged proteins are preferentially partitioned into one daughter cell, preserving the integrity of the other. In stem cells, this mechanism may help balance self-renewal with commitment, whereby the daughter cell carrying the proteotoxic load is lost to terminal differentiation, thus maintaining the functional integrity of the stem cell pool [116,117]. HSCs can use a similar mechanism to offload damaged proteins and organelles to daughter cells exiting the stem cell pool [9,14,114]. New insights suggest that this process becomes less efficient with age [118]. CASIN, an inhibitor of the GTPase Cdc42 enhances HSC polarity and the capacity for asymmetric distribution of subcellular content [119,120]. In preclinical studies, Cdc42 inhibitors demonstrated promising results, potentially presenting a novel way to improve stem cell fitness and lifespan [121–123].

While these interventions aim to clear or reallocate damaged proteins, a defining feature of adult HSCs is their intrinsically low rate of protein translation, a trait shared by many adult stem cells [124]. By contrast, fetal HSCs are highly proliferative and exhibit elevated translation to support rapid expansion during development [13,125]. Bile acids, which act as chemical chaperones and prevent protein aggregation, may help explain why the liver serves as a key site of fetal hematopoiesis [126]. On the other hand, the liver also supports quiescence in adult HSCs, serving as the primary source of thrombopoietin, a cytokine essential for maintaining HSC dormancy [127–129]. The vital example of the liver at both developmental extremes underscores that systemic factors beyond the bone-marrow niche can shape HSC function, highlighting the limitations of studying these cells in isolation. In addition to plasma-derived signals such as bile acids, cytokines, and metabolites, cues from the central nervous system have been shown to influence distal organs and modulate proteostasis in peripheral tissues, at least in simpler model organisms [130]. Together, these observations support the emerging view that proteostasis in HSCs is not governed solely by cell-intrinsic mechanisms but is integrated into a broader physiological network influenced by developmental context and systemic signals.

In no other hematopoietic compartment is the disconnect between transcription and translation as pronounced as in HSCs [39]. This translational restraint may help maintain low protein synthesis—either through altered ribosomal activity or by storing a substantial pool of mRNAs in a repressed state, poised for rapid translation upon differentiation [62]. Elevated translation, by contrast, is associated with a shorter lifespan, potentially because higher protein production increases the risk of misfolding and proteotoxicity or diverts critical energy and building blocks from repair processes [76]. Adding further complexity, ribosomal subunits have functions beyond protein translation, including roles in metabolic control and even in protein degradation by releasing ubiquitin molecules from ribosomal precursor peptides [131,132]. HSCs exhibit tight ribosomal restraint, a feature thought to support their longevity, and understanding these functions—along with the ribosomal stress response—may uncover broader strategies for promoting cellular health span.

## Concluding remarks

Given its stress resilience and the diversity of protein quality control mechanisms among its cell types, the blood provides a powerful system for dissecting proteostasis regulation. Both cellular and soluble blood components have been shown to influence organismal aging and tissue rejuvenation, though some findings remain controversial [24,25,84,85,133]. Compared with their immediate progeny—multipotent progenitor cells—HSCs exhibit markedly lower protein synthesis uncoupled from transcript levels [37,39], reduced proteasomal activity [111], and greater reliance on basal autophagy [134], although lysosomal sequestration does not always result in complete digestion [135]. HSCs also display a distinct chaperone and stress-response profile optimized to maintain protein fidelity under conditions of minimal translation [50].

Despite substantial progress, key questions remain. Future research should focus on the understudied roles of ribosomal and nucleolar checkpoints in HSC fate decisions, which may hold untapped therapeutic potential. Ribosomes are now recognized not only as translational machinery but also as regulators of cellular viscosity, nuclear stress, and aging, underscoring the need to investigate the fate-determining functions these complexes may hold.

Another promising direction is the role of mitochondrial proteases in regulating proteins beyond their immediate environment, potentially affecting cytosolic and nuclear processes through mechanisms still poorly understood. In HSCs, at least three mechanisms have been proposed to restrain mitochondrial activity and minimize metabolic damage: mitophagy, lysosomal sequestration, and functional suppression [88,106,110]. While the pathways may overlap to some degree, partially conflicting findings across these three studies highlight the need for robust, standardized experimental models. Clarifying these mechanisms is important, as it may reveal new targets for metabolic regulation and the development of anti-aging interventions.

*Ex vivo* organoid systems mimicking the bone-marrow niche may enable long-term cell tracking and new insights, but current models fail to preserve true HSC quiescence and instead induce proliferation [136]. Overcoming this limitation is both a technical challenge and a necessary step forward.

Future studies should emphasize subtle, physiologically relevant perturbations rather than extreme genetic or pharmacologic interventions, which often trigger apoptosis and mask more nuanced regulatory mechanisms. Focusing on incremental, context-specific stressors may better illuminate how proteostasis is regulated during aging.

In conclusion, this review highlights how HSCs preserve proteostasis to sustain longevity. Addressing remaining gaps will require interdisciplinary approaches that integrate biochemical, genetic, and systems-level analyses (see Outstanding questions), ultimately enabling new strategies to bolster stem cell resilience and promote healthy aging.

## Outstanding questions

Selective vulnerability in the proteome: are certain classes of proteins—such as intrinsically disordered proteins, components of biomolecular condensates, or DNA/RNA-binding proteins with basic charge—more susceptible to misfolding and aggregation during aging and stress? Identifying which protein types are preferentially translated across hematopoietic stages may uncover specific vulnerabilities and compensatory mechanisms in proteostasis.

Mitochondrial content versus activity: a recent study has demonstrated a decoupling of mitochondrial mass from metabolic activity in HSCs. The enrichment of mitochondria in highly functional, aged HSCs suggests that these organelles are maintained in a low-activity state, potentially primed for reactivation upon differentiation. The increase in mitochondrial mass during aging may reflect an adaptive response, and that mitochondria can be restrained through mechanisms other than lysosomal sequestration or digestion.

Reversibility of sequestration mechanisms: how reversible are protein sequestration mechanisms in HSCs, and does aging shift the balance between dynamic condensation and irreversible aggregation? Biomolecular condensates play key roles in regulating translation and proteostasis, but their composition and dynamics in HSCs remain poorly defined. Age may alter the formation and resolution of these condensates. Similarly, while lysosomes are typically terminal degradation sites, the persistence of unacidified vacuoles in quiescent HSCs suggests a potential for selective recovery of content. Elucidating how age affects these compartmentalization strategies will be critical for understanding the adaptability of proteostasis in stem cells.

Systemic control of stem cell proteostasis: to what extent do extrinsic cues such as neuronal signals or blood-borne factors modulate HSC proteostasis? Understanding how these systemic influences regulate protein stress could open therapeutic avenues for enhancing stem cell resilience and longevity.

Physiological versus supraphysiological perturbation: many experimental approaches rely on severe interventions that may not reflect native stress responses. Future work should prioritize graded, physiologically relevant perturbations to reveal regulatory networks that operate under homeostatic or mildly stressful conditions.

Modeling quiescence *in vitro*: despite advances in HSC expansion, current *in vitro* systems fail to preserve true quiescence. Developing mouse and human models that maintain low metabolic and translational activity will be essential for studying physiological proteostasis and for expanding functional, non-exhausted human HSCs for clinical use.

## Highlights

The hematopoietic system represents an excellent model to study how proteostasis (protein homeostasis) influences different cell types within the same tissue. This review focuses on mechanisms of proteostasis that preserve the lifespan of rare hematopoietic stem cells (HSCs).

Although most proteostasis network components are expressed in all cells, their activation and utilization are cell-type-specific.

HSCs maintain low translation rates and a preference for autophagy over proteasomal degradation to minimize protein stress.

To protect the integrity of the stem cell pool, HSCs are thought to respond to damage by clearing defective organelles and proteins or by eliminating compromised cells through differentiation or apoptosis.

A stressed proteome accelerates HSC aging, and the immune system derived from aged HSCs is suspected to contribute to the decline of other tissues. This highlights the importance of maintaining healthy HSCs to preserve organismal wellbeing.

Several experimental treatments in mouse models have been shown to boost HSC activity in older organisms by enhancing proteostasis. This promising research opens up new possibilities for interventions that could improve aging through regenerative medicine.
